# Structural Study of Agmatine Iminohydrolase From *Medicago truncatula*, the Second Enzyme of the Agmatine Route of Putrescine Biosynthesis in Plants

**DOI:** 10.3389/fpls.2019.00320

**Published:** 2019-03-28

**Authors:** Bartosz Sekula, Zbigniew Dauter

**Affiliations:** Synchrotron Radiation Research Section of Macromolecular Crystallography Laboratory, National Cancer Institute, Argonne, IL, United States

**Keywords:** polyamine biosynthesis, putrescine, beta/alpha propeller fold, penteins, agmatine deiminase, guanidine-modifying enzymes

## Abstract

Plants are unique eukaryotes that can produce putrescine (PUT), a basic diamine, from arginine *via* a three-step pathway. This process starts with arginine decarboxylase that converts arginine to agmatine. Then, the consecutive action of two hydrolytic enzymes, agmatine iminohydrolase (AIH) and *N-*carbamoylputrescine amidohydrolase, ultimately produces PUT. An alternative route of PUT biosynthesis requires ornithine decarboxylase that catalyzes direct putrescine biosynthesis. However, some plant species lack this enzyme and rely only on agmatine pathway. The scope of this manuscript concerns the structural characterization of AIH from the model legume plant, *Medicago truncatula*. *Mt*AIH is a homodimer built of two subunits with a characteristic propeller fold, where five αββαβ repeated units are arranged around the fivefold pseudosymmetry axis. Dimeric assembly of this plant AIH, formed by interactions of conserved structural elements from one repeat, is drastically different from that observed in dimeric bacterial AIHs. Additionally, the structural snapshot of *Mt*AIH in complex with 6-aminohexanamide, the reaction product analog, presents the conformation of the enzyme during catalysis. Our structural results show that *Mt*AIH undergoes significant structural rearrangements of the long loop, which closes a tunnel-shaped active site over the course of the catalytic event. This conformational change is also observed in AIH from *Arabidopsis thaliana*, indicating the importance of the closed conformation of the gate-keeping loop for the catalysis of plant AIHs.

## Introduction

Biosynthesis of putrescine (PUT) starts from arginine (ARG) and follows one of the two pathways which comprise agmatine (AGM) or ornithine (ORN) biotransformation (Michael, 2017). The AGM route is important and widely spread among plants, algae, and prokaryotic organisms (Michael, 2016). First, ARG is decarboxylated to AGM by arginine decarboxylase (ADC). The later conversion of AGM to PUT in plants is carried out by agmatine iminohydrolase (AIH) and further by *N-*carbamoylputrescine amidohydrolase (CPA), an octameric protein with a quaternary structure resembling an incomplete left-handed helix (Sekula et al., 2016). *AIH* and *CPA* genes have been acquired by plants through an endosymbiotic gene transfer from the cyanobacterial ancestor of the chloroplast (Illingworth et al., 2003). Actually, plants are unique eukaryotes to biosynthesize PUT *via* the AGM biotransformation, which makes ADC, AIH, and CPA potential targets for herbicide design (Böger and Sandmann, 1989). In some bacteria, AGM-to-PUT conversion is also catalyzed by agmatine ureohydrolase (agmatinase) (Satishchandran and Boyle, 1986) and most of the cyanobacteria use this enzyme instead of AIH and CPA (Fuell et al., 2010). Some Gram-positive bacteria may also obtain PUT in a catabolic pathway which produces ATP from the carbamoyl phosphate obtained from the AGM-to-PUT transformation (Llacer et al., 2007). The second manner of PUT biosynthesis, the ORN route, is dominant for most eukaryotes, including animals and fungi, and involves ORN decarboxylation catalyzed by ornithine decarboxylase (ODC) (Janowitz et al., 2003). Some plant species like *Arabidopsis thaliana* and *Physcomitrella patens* do not have the *ODC* gene (Hanfrey et al., 2001) and they rely only on the AGM-to-PUT bioconversion. Other plants, which have preserved *ODC*, may obtain PUT either from AGM or ORN.

PUT is the starting backbone for larger polyamines (PAs) which are produced by specialized aminopropyltransferases, enzymes which use decarboxylated S-adenosylmethionine as a donor of the aminopropyl group. Therefore, the first transfer of the aminopropyl group to PUT, catalyzed by spermidine synthase (SPDS), yields triamine spermidine (SPD). SPD is the substrate for the second transfer which yields symmetrical spermine or unsymmetrical thermospermine. For a long time, it was elusive whether both tetraamines are biosynthesized in plants, but they are actually formed by two distinct proteins, spermine synthase (SPMS) and thermospermine synthase (TSPS). Aminopropyltransferases are distinguished by several structural features that favor each enzyme toward the specific PA production (Sekula and Dauter, 2018).

PAs are essential for the regulation of various physiological processes which secure the proper growth and development of higher plants (Takano et al., 2012; Jimenez-Bremont et al., 2014; Minocha et al., 2014; Tiburcio et al., 2014; Liu et al., 2015). The cationic character of PAs promotes their interactions with anionic proteins and nucleic acids, thus affecting transcription, translation (Gill and Tuteja, 2010; Igarashi and Kashiwagi, 2010; Tiburcio et al., 2014), and the rate of membrane transport (Pottosin et al., 2014; Pottosin and Shabala, 2014). SPD is an important donor of aminobutyl group for the posttranslational modification of the hypusine-containing translation elongation factor eIF5A in eukaryotes and archaea (Prunetti et al., 2016). PAs can also modulate the activity of antioxidant enzymes and thereby influence the concentration of reactive oxygen species (Radhakrishnan and Lee, 2013; Kamiab et al., 2014; Mostofa et al., 2014). PA accumulation is often related with its protective role for the environmental stress conditions and leads to an increase of stress tolerance of the plant (Capell et al., 2004; Alcazar et al., 2010; Wang et al., 2011; Berberich et al., 2015). Meanwhile, defects of PA biosynthesis pathway result in the retardation, sterility, and other developmental pathologies in plants (Hanzawa et al., 2000).

Herein, we describe the structural characterization of AIH from *Medicago truncatula* (*Mt*AIH), the model legume plant. The enzyme is responsible for the second step of the AGM pathway of PUT biosynthesis, that is the hydrolytic conversion of AGM to *N-*carbamoylputrescine (NCP) with the release of ammonia. Plant AIHs, as well as CPAs, do not contain chloroplast-targeting peptides and they act in the cytoplasm. This is opposite to the first enzyme of the pathway, ADC, which initializes PUT biosynthesis in plastids (Illingworth et al., 2003). The advantage of AGM production outside plastids could be explained by the availability of AGM in the cytoplasm not only for PUT production but also for the biosynthesis of *N*-hydroxycinnamoyl conjugates, which may serve as precursors of defensive compounds (Burhenne et al., 2003). AIH belongs to one of the seven types of guanidine-modifying enzymes (GMEs) (Shirai et al., 2006). It is a member of the pentein superfamily that is characterized by the propeller-like arrangement of five repeated motifs that form a narrow channel with a central, negatively charged core (Hartzoulakis et al., 2007). The conserved catalytic triad of GMEs (His, Asp, and Cys) is responsible for a range of activities, which cover transferase and hydrolytic reactions on the guanidine-containing compounds (Hartzoulakis et al., 2007). Although AIHs from various plant species, including corn (Yanagisawa and Suzuki, 1981), soybean (Park and Cho, 1991), and maize (Yanagisawa, 2001) were isolated, there is no published structural characterization of any plant AIH available, except for unpublished reported entries in the Protein Data Bank (PDB) of AIH from *A. thaliana* (*At*AIH, PDB ID 3H7K, 3H7C, 1VKP, Center for Eukaryotic Structural Genomics).

In this work, we present the high-resolution crystal structure of non-liganded *Mt*AIH and the structure with the reaction product analog—6-aminohexanamide (AHX). This, combined with the in-solution small-angle X-ray scattering analysis, provides data for the characterization of this plant AIH and a detailed comparison of plant AIHs (*Mt*AIH and *At*AIH) with their bacterial orthologs.

## Materials and Methods

### Cloning, Overexpression, and Purification of *MtAIH*

In order to express and purify *Mt*AIH (UniProt ID G7JT50), we used the protocol which was recently successfully applied in the studies of other plant enzymes (Ruszkowski et al., 2018; Sekula et al., 2018). Briefly, the following primers, forward: TACTTCCAATCCAATGCCCATGGCTTTCACATGCCTGCAGAAT and reverse: TTATCCACTTCCAATGTTACTAAATGGCTGGTTGTTGCTGAGTGAT and the cDNA from leaves of *M. truncatula* as a template were used in a polymerase chain reaction (PCR) prior to obtaining *Mt*AIH open reading frame (MTR_4g112810) with encoded protein starting from codon number 11. The incorporation of *Mt*AIH gene into the pMCSG68 vector (Midwest Center for Structural Genomics) was performed according to the ligase-independent cloning (Kim et al., 2011) protocol. The vector introduces an N-terminal His_6_-tag followed by the Tobacco Etch Virus (TEV) protease cleavage site to the cloned protein and the Ser-Asn-Ala linker that is not cleaved from the expressed protein. In the next step, the BL21 Gold *E. coli* competent cells (Agilent Technologies) were transformed with the vector containing the *Mt*AIH gene. The cells were precultured at 37°C in LB medium with the addition of ampicillin (150 μg/ml) overnight. Next, 1.5% v/v of the culture was used as the inoculum of the fresh LB medium with ampicillin. It was cultured at 37°C until OD_600_ reached a value 1.0. In the next step, the culture was cooled to 10°C for 2 h and then the protein expression was induced with 0.5 mM of isopropyl-β-D-thiogalactopyranoside (IPTG). The protein overexpression was carried out at 18°C for 16 h. Before pelleting the cells in the centrifuge at 3,500 × g for 30 min, the culture was cooled to 4°C. Cell pellets were resuspended in 35 ml of the binding buffer [50 mM HEPES pH 7.4; 500 mM NaCl; 20 mM imidazole; 1 mM tris(2-carboxyethyl)phosphine, TCEP] and frozen at −80°C. Thawed cells were disrupted by sonication in an ice/water bath for 4 min (bursts of 4 s with 26-s intervals). Then, the cellular debris was pelleted by centrifugation at 25,000 × g for 30 min at 4°C.

The first step of *Mt*AIH purification was performed on a column packed with 5 ml of HisTrap HP resin (GE Healthcare) connected to the Vac-Man laboratory vacuum manifold (Promega). The supernatant was applied to the column and washed five times with 40 ml of the binding buffer. The protein elution was performed with 20 ml of the elution buffer (50 mM HEPES pH 7.4; 500 mM NaCl; 400 mM imidazole; 1 mM TCEP). His_6_-tagged TEV protease (final concentration of 0.1 mg/ml) was used to cleave the His_6_-tag from *Mt*AIH. This step was simultaneous to the overnight dialysis at 4°C against the dialysis buffer (50 mM HEPES pH 8.0; 500 mM NaCl; 1 mM TCEP). After dialysis, the sample was applied on HisTrap HP resin to remove the cleaved His_6_-tag and His_6_-tagged TEV protease. The final step of the purification of *Mt*AIH was size exclusion chromatography on HiLoad Superdex 200 16/60 column (GE Healthcare) connected to an AKTA FPLC system (Amersham Biosciences). The column was equilibrated in 50 mM HEPES pH 7.4, 100 mM KCl, 50 mM NaCl, and 1 mM TCEP.

### Crystallization and Data Collection

*Mt*AIH was concentrated with Amicon concentrators (Millipore) to the final concentration of 8 mg/ml, determined by the absorbance measurement at 280 nm with the extinction coefficient of 77,920. The composition of the protein buffer was the same as the buffer used for the size exclusion chromatography. The sample was subjected to crystallization trials with use of Morpheus Screen (Molecular Dimensions) and PEG/Ion Screen (Hampton Research). Unused protein was stored in −80°C in 50-μl aliquots for later use. Crystals of *Mt*AIH were grown in 0.2 M sodium acetate, 20% PEG 3350 at pH 8.0. The *Mt*AIH-AHX complex was obtained by cocrystallization of *Mt*AIH with 10 mM of the ligand in 37th conditions of Morpheus Screen (Molecular Dimensions; 0.12 M Alcohols, 0.1 M Buffer System 1 at pH 6.5, 30% v/v Precipitant Mix 1) diluted with water to 70% of original concentration. Glycerol (25%) was used as a cryoprotectant for the freezing of native crystals. Crystals of *Mt*AIH-AHX were cryoprotected by original 37th conditions of Morpheus Screen. Protein was crystallized by sitting and hanging drop methods.

Diffraction data were collected at SER-CAT 22-ID beamline at the Advanced Photon Source (APS), Argonne National Laboratory, USA. The data were processed with *XDS* (Kabsch, 2010) and scaled using anisotropic diffraction limits with *STARANISO*^1^. The anisotropic cut-off surface for *Mt*AIH data has been determined with best and worst diffraction limits 1.20 and 1.42 Å, respectively. In the case of *Mt*AIH-AHX, the diffraction resolution was truncated between 2.20 and 2.66 Å. Table 1 provides detailed statistics for spherical and anisotropic truncation. Anisotropic data treatment improved the electron density maps of refined structures. Coordinates and structure factors were deposited in the PDB with the following IDs: 6NIB (*Mt*AIH), 6NIC (*Mt*AIH-AHX).

**Table 1:** Data-collection and refinement statistics.

Structure	*Mt*AIH	*Mt*AIH-AHX
**Data collection**		
Beamline	22-ID	22-ID
Wavelength (Å)	1.00	1.00
Temperature (K)	100	100
Oscillation range (°)	0.5	0.5
Space group	*C*2	*P*6_1_22
Unit cell parameters (Å,°)	*a* = 147.0 *b* = 75.5 *c* = 47.1, *β* = 108.6	*a* = *b* = 142.4 *c* = 345.4
Resolution^1^ (Å)	39.96–1.20^2^ (1.28–1.20)	50.19–2.20^3^ (2.38–2.20)
Reflections collected/unique	440,225/121248 (17,439/5584)	634,341/80033 (28,294/4106)
Completeness (%)		
Spherical Ellipsoidal	79.2 (19.5) 93.8 (58.5)	76.7 (19.3) 94.7 (68.2)
Multiplicity	3.6 (3.1)	7.9 (6.9)
*R*_merge_ (%)	3.1 (46.3)	12.1 (72.6)
<*I*/σ(*I)*>	19.1 (2.5)	13.1 (2.7)
*CC_1/2_* (%)	100 (75.8)	99.8 (81.4)
**Refinement**		
*R_free_* reflections	1,568	1,232
No. of atoms (non-H)		
Protein	2,898	11,351
Ligands	34	80
Solvent	587	773
*R*_work_/*R*_free_ (%)	11.2/13.7	16.5/20.4
Mean ADP^4^ (Å^2^)	10.4	33.8
RMSD from ideal geometry		
Bond lengths (Å)	0.014	0.018
Bond angles (^o^)	1.4	1.9
Ramachandran statistics (%)		
Favored	98	96
Allowed	2	4
Outliers	0	0
PDB code	6NIB	6NIC

^1^Best anisotropic diffraction limit cut-off.

^2^Worst diffraction limit after cut-off is 1.42 Å.

^3^Worst diffraction limit after cut-off is 2.66 Å.

^4^ADP, atomic displacement parameter. Values in parentheses refer to the highest resolution shell.

### Structure Determination and Refinement

The structure of *Mt*AIH was solved by molecular replacement in *Phaser* (McCoy et al., 2007) with the structure of *At*AIH (PDB ID 1VKP) as a search model. The initial model was rebuilt in *PHENIX AutoBuild* (Terwilliger et al., 2008). Then, the structure was subjected to manual and automatic refinement with *Coot* (Emsley et al., 2010) and *Phenix* (Adams et al., 2010) with anisotropic *B*-factors. Refined structure of unliganded *Mt*AIH was used as a model for determination of the structure of *Mt*AIH-AHX that was refined with isotropic *B-*factors and *TLS* (Winn et al., 2001, 2003) in *Refmac* (Murshudov et al., 2011). The refinement was carried out until the *R_work_* and *R_free_* values (Brunger, 1992), the geometric parameters, and the overall difference electron density maps were satisfactory. Evaluation of the final structures was performed in *PROCHECK* (Laskowski et al., 1993) and *MolProbity* (Chen et al., 2010). The final refinement statistics are given in Table 1.

### Small-Angle X-Ray Scattering Data Collection and Analysis

**Figure 1:**
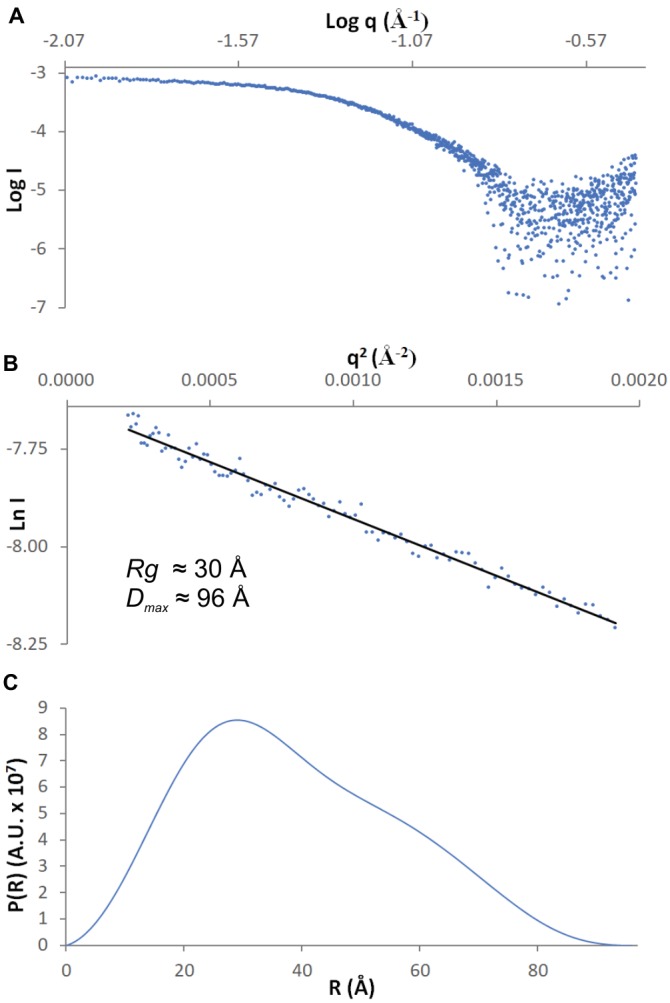
SAXS data. **(A)** The experimental curve for *Mt*AIH; **(B)** Guinier plot (blue dots) of the scattering curve with the best fit shown as a black line; **(C)** Pair-distance distribution function for *Mt*AIH SAXS data.

SAXS data were collected from 5.5 mg/ml *Mt*AIH solution at the BioCAT 18-ID beamline (Fischetti et al., 2004) at APS with in-line size exclusion chromatography (SEC-SAXS) to separate sample from aggregates, thus ensuring optimal sample homogeneity. The sample was loaded on a WTC-015S5 column (Wyatt Technologies) connected to an Infinity II HPLC (Agilent Technologies). The sample after the column was sent to the Agilent UV detector, a Multi-Angle Light Scattering (MALS) detector, and a Dynamic Light Scattering (DLS) detector (DAWN Helios II, Wyatt Technologies), and an RI detector (Optilab T-rEX, Wyatt). Molecular weights and hydrodynamic radii were calculated from the MALS and DLS data respectively using the ASTRA 7 software (Wyatt). Afterward, the sample was sent to the SAXS flow cell, a 1.5-mm quartz capillary. Scattering intensity was recorded at 1.03-Å wavelength at room temperature, with 0.5-s exposures every 2 s on a Pilatus3 1M detector (Dectris) placed 3.5 m from the capillary (collected q-range was 0.004–0.4 Å^−1^). Data reduction and analysis were performed by *BioXTAS RAW* 1.5.1 (Hopkins et al., 2017). Frames corresponding to the elution peak of the chromatogram were averaged to maximize the signal-to-noise ratio. Several frames immediately proximal to the sample peak (buffer frames) were averaged and subtracted from the sample scattering to obtain the final SAXS curve (Figure 1A). The *Rg* value calculated from the Guinier (Figure 1B) and distance distribution analysis (Figure 1C) was 30 Å. The calculated maximum dimension of the particle (*D*_max_) was 96 Å. The *qRg* limits for further calculations were 0.43–1.29.

*Ab initio* envelopes with the restraint of twofold symmetry were calculated in *DAMMIF* (Franke and Svergun, 2009), averaged with *DAMAVER* (Volkov and Svergun, 2003), refined with *DAMMIN* (Svergun, 1999), and filtered with *DAMFILT. SUPCOMB* was used for the superposition of the SAXS envelope with the crystallographic dimer of *Mt*AIH. *DENSS* (Grant, 2018) was used for the calculation of the *ab initio* electron density maps directly from the SAXS data with no prior information about the symmetry of the molecule.

### Other Software Used

Molecular illustrations were created with UCSF *Chimera* (Pettersen et al., 2004). Ramachandran plot was calculated in *Rampage* (Lovell et al., 2003). Secondary structure was recognized with *ProMotif* (Hutchinson and Thornton, 1996) within the *PDBsum* server (de Beer et al., 2014). Sequence alignments were performed in *CLUSTAL W* (Thompson et al., 1994) and edited in *BioEdit* (Hall, 1999).

## Results and Discussion

### *Mt*AIH Presents the Pentein α/β Propeller Fold

**Figure 2:**
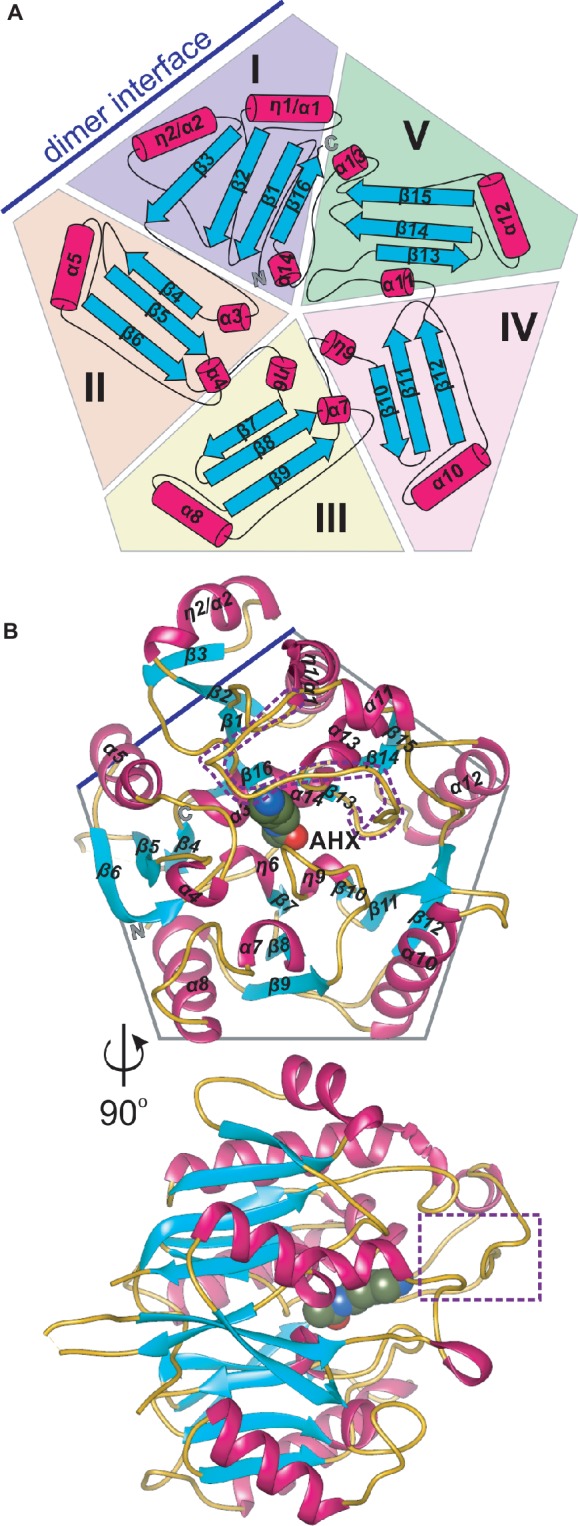
The monomer of *Mt*AIH. **(A)** Topological diagram of *Mt*AIH with a schematic depiction of the five αββαβ units (I–V) that form internal pseudo fivefold symmetry; secondary structure elements, helices (cylinders) and sheets (arrows) are colored in red and cyan, respectively; blue line indicates the position of the second *Mt*AIH subunit from the dimeric assembly. **(B)** Ribbon representation of the structure of *Mt*AIH monomer in complex with AHX (shown as spheres); the gate-keeping loop over the active site is marked with a purple dotted line.

AIHs are assigned by the Structural Classification of Proteins (SCOPe) (Fox et al., 2014) to the porphyromonas-type peptidylarginine deiminase family that is a part of the Superfamily of penteins, characterized by a propeller-like arrangement of five αββαβ units which form a narrow channel in the core (Hartzoulakis et al., 2007). Penteins share a conserved group of residues that recognize the guanidine moiety of the substrate—His, Cys, and two acidic, guanidine-binding residues (usually Asp) which serve to catalyze a range of reactions (Linsky and Fast, 2010). The monomer of *Mt*AIH is no different in this matter, i.e., five motif repeats (I–V) are arranged around fivefold pseudosymmetry axis that is aligned with the catalytic tunnel in the core of the protein (Figures 2A,B). Class, Architecture, Topology, Homology (CATH) server (Sillitoe et al., 2015) matches *Mt*AIH with the L-arginine/glycine amidinotransferase superfamily that belongs to the Class 3 of alpha beta proteins with the architecture of a five-bladed propeller.

The overall globular shape of the *Mt*AIH monomer resembles pentagonal prism, where the longest helices (η1/α1, α1, α7, α9, α11) are positioned in the imaginary vertices of the pentagon with all β-strands running along the direction marked by these helices. Sixteen strands in *Mt*AIH form five β-sheets, one four-stranded, and four with three strands each. All β-sheets are oriented toward the center of the molecule forming five “blades” of the propeller (Figure 2B). Repeat I with its four-stranded β-sheet actually disturbs the overall fivefold pseudosymmetry of the molecule, i.e., it has the additional strand β3 and the helix η2/α2 which are placed outside the pentagonal shape. Moreover, the αββαβ motif of unit I is, in fact, discontinuous and it is fully formed with the complementation of C-termini, more precisely, by α14 and β16 (Figure 2A). In the center of the molecule, four short helices (α3, η6, η9, α14), that directly precede internal strands from repeats I–IV, line the surface of the negatively charged central channel, that is the active site. These core helices are placed on N-terminal sides of the inner strands of β sheets from repeats I–IV. Only the inner strand of repeat V (β13) is not directly preceded by a short helix. Instead, the first helix of this repeat (α11) is actually longer than helices that build the active site and it is placed almost outside of the outline of the protein which precludes it from the interactions with the substrate in the active site. Additionally, α11 is flanked by long coils. One of these coils (residues 291–314) covers the active site entrance and plays a crucial role in the substrate recognition (see below for details). *Mt*AIH has a very high structural similarity to the other plant ortholog, *At*AIH (unpublished, PDB ID 3H7K, overall sequence identity is 70%), with the 0.6 Å root mean square deviation of the superposed structures. Both plant AIHs have a similar organization of secondary structure and almost identical architecture of the active site.

### *Mt*AIH Forms Symmetric Dimers

**Figure 3:**
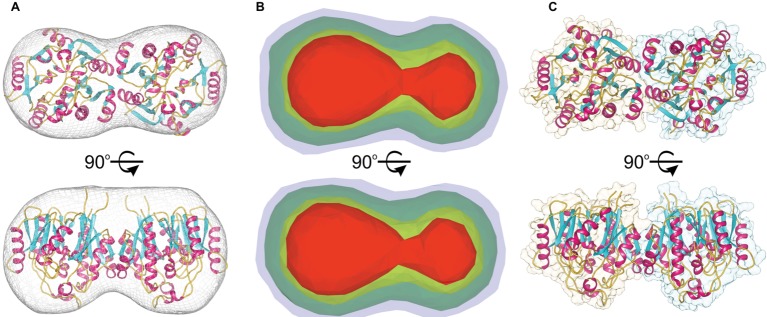
*Mt*AIH dimers. **(A)** *Ab initio* SAXS envelope (gray mesh) with the superposed crystallographic dimer of *Mt*AIH; **(B)** *Ab initio* electron density maps of *Mt*AIH calculated by *DENSS* from the SAXS data; contours of the map are as follows: 5σ (red), 3.5σ (yellow), 1.4σ (green), 0.7σ (blue). **(C)** The shape of the crystallographic dimer in the *At*AIH structure (PDB ID 1VKP).

The molecular mass of *Mt*AIH calculated from MALS is 81 kDa, which almost ideally agrees with the molecular weight of two monomers (theoretical mass of the *Mt*AIH construct is 41.2 kDa). The dimeric assembly of *Mt*AIH is also shown by SAXS envelope even though the calculated mass of *Mt*AIH from SAXS was underestimated to be ~60 kDa. The envelope with twofold symmetry restraints (Figure 3A) and *ab initio* electron density map that was calculated with no information about the molecule symmetry (Figure 3B) clearly correspond to the *Mt*AIH dimer in the crystal lattice where two subunits are related by twofold symmetry. It is worth to note that both crystal structures, *Mt*AIH and *Mt*AIH-AHX, present different crystallographic symmetry (see Table 1) with one and four chains in the asymmetric unit, respectively. In the case of the unliganded structure, the dimer is created by a crystallographic twofold axis, while in the *Mt*AIH-AHX complex, there are two almost identical dimers in the asymmetric unit. The comparison of our results with the crystal structure of *At*AIH (Figure 3C) clearly shows that *At*AIH also forms dimers analogical to *Mt*AIH.

The results are somewhat contrary to the analysis of the *Mt*AIH crystal structure done with the PISA server (Krissinel and Henrick, 2007). It shows that monomer of *Mt*AIH has the surface area ~14,000 A^2^ with the biggest interface area ~900 Å^2^ that is shared with the closest monomer in the crystal lattice. It is about 6.5% of the monomer surface and it was estimated by the PISA server to have no role in the complex formation. However, in both *Mt*AIH crystal forms, this interface area between two subunits is preserved and, taking it together with SAXS and MALS results, it is, in fact, responsible for the formation of *Mt*AIH dimers. The intersubunit interactions on this interface involve 25 residues (the same set from both interacting subunits) where about half of them create 20 hydrogen bonds or salt bridges. The interface residues belong to the β2, β3, and η2/α2 from repeat I and α5 which belongs to the repeat II (Figure 2B). Two subunits of *At*AIH in the crystal structure (PDB ID 3H7K) share the analogical interface area and the PISA server analysis does not recognize it as a dimer either. Most of the interacting residues are preserved in both plant orthologs, but *At*AIH evidently presents fewer interacting residues which altogether form only 14 hydrogen bonds.

**Figure 4:**
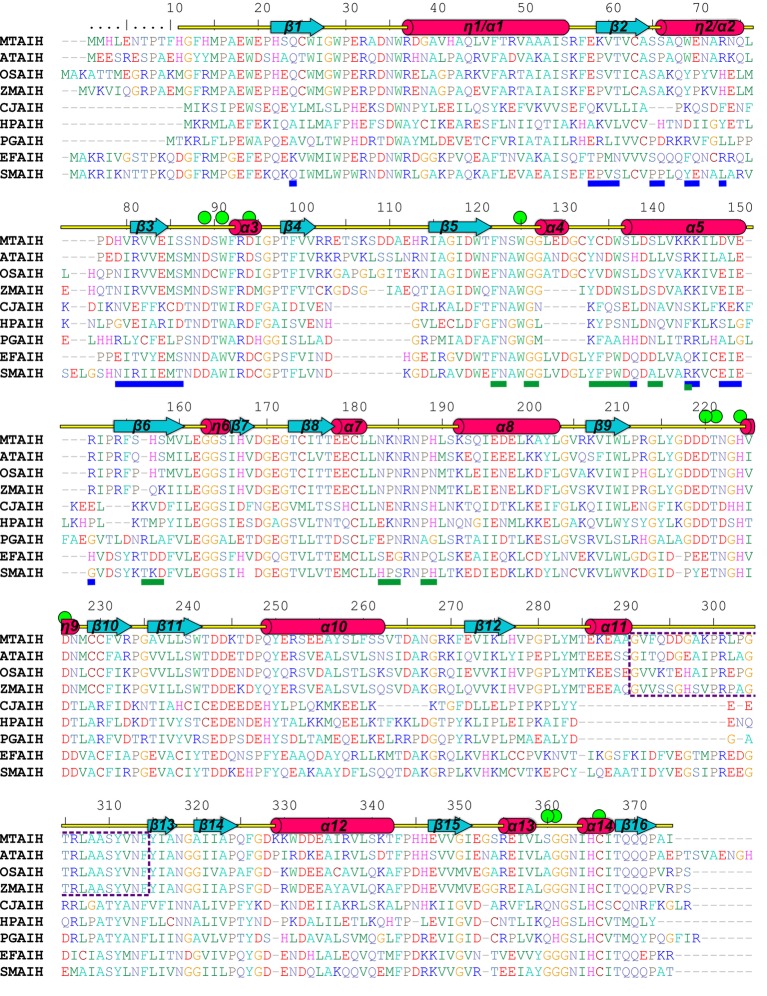
Sequence alignment of selected AIHs. The alignment was made with the following AIH sequences (*UniProt* accession numbers are given in square brackets, as well as the sequence identity with *Mt*AIH): *Mt*AIH [G7JT50], *At*AIH [Q8GWW7, 70% sequence identity], *Os*AIH [Q01KF3, 65%], *Zm*AIH [C0PHP8, 64%], *Cj*AIH [Q0P9V0, 26%], *Hp*AIH [O24890, 26%], *Pg*AIH [Q7MXM8, 27%], *Ef*AIH [Q837U5, 44%], *Sm*AIH [Q8DW17, 45%]. Sequence positions above the alignment and annotation of the secondary structure elements (α helices and 3_10_ helices, η, are shown as red cylinders and β strands are shown as cyan arrows) correspond to *Mt*AIH. Residues are color-coded by type. Green circles indicate residues that form the active site or participate in the interactions with the bound substrate. Blue and green lines below the alignment indicate residues that form dimer interface in *Mt*AIH and *Hp*AIH, respectively. The gate-keeping loop over the active site of plant AIHs is marked with a purple dotted line.

A dimeric assembly was independently reported for the other plant AIHs, including *At*AIH (Janowitz et al., 2003), AIH from maize (*Zm*AIH) (Yanagisawa, 2001) and rice (*Os*AIH) (Mohan Chaudhuri and Ghosh, 1985). The reported exception (Park and Cho, 1991) is a 70-kDa monomeric protein from soybean that was described to have AIH activity. However, the authors did not provide the sequence of the isolated protein. Also, any record classified as AIH matches to the reported description. The sequence alignment (Figure 4) of the dimeric plant AIHs shows that 19 residues (out of the pool of 25 which form the interface in *Mt*AIH) are identical or very similar in all four plant AIHs. A similar extent of conservation applies to polar and hydrophobic residues on the dimer interface. Identical polar positions are: Gln24, Glu58, Thr61, Ser65, Gln68, Arg73, Arg81, Glu84, Ser86 Lys145, Glu150, and Arg151. These residues in *Mt*AIH are involved in 14 hydrogen bonds, that is, 70% of all hydrogen bonds found in the interface analysis.

**Figure 5:**
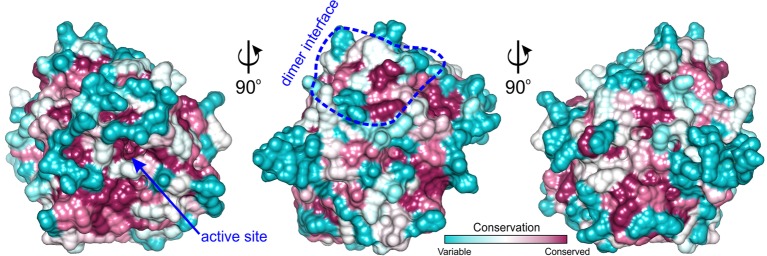
*ConSurf* analysis for *Mt*AIH. Surface representation of the *Mt*AIH monomer where surface residues are color-coded by the conservation score calculated from the sequence alignment of plant AIHs; the analysis was done in *ConSurf* (Ashkenazy et al., 2016); dimer interface is circled in blue. The pool of 184 plant AIH sequences was selected from the protein sequences classified to agmatine deiminase family (IPR017754) by *InterPro* (Finn et al., 2017); clear outliers where the sequence length was shorter than 340 or longer than 420 were manually excluded from the alignment.

Analyzing sequence conservation of all plant AIHs (Figure 5), there is no obvious highly conserved area around the dimer interface that can be distinguished right away. However, when considering the conservation of particular residues involved in the hydrogen bonding between both subunits, most of them stand out as highly conserved (Val60, Arg73, Arg81, Val82, Glu84, Ser86, Lys145), whereas only three are very variable residues (Asp148, Val149, Arg151). Moreover, hydrophobic interactions seem to be important for the dimer formation as well—at the center of the interface between dimer mates there is a patch of apolar residues (Trp69, Val83, Ile85, Val149). All of these residues are apolar in plant AIHs.

### Bacterial AIH Analogs With Various Biological Assemblies

**Figure 6:**
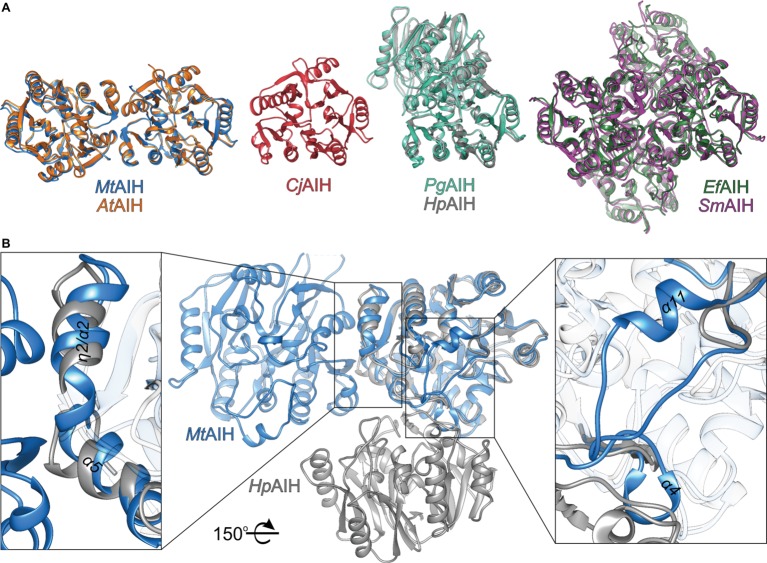
Comparison of AIHs from different species. **(A)** Various assemblies of AIHs (starting from the left): dimeric plant AIHs (*At*AIH, orange, and *Mt*AIH, blue), bacterial monomeric AIH (*Cj*AIH, red), dimeric AIHs (*Pg*AIH, light green, *Hp*AIH, gray), and tetrameric AIHs (*Ef*AIH, dark green, and *Sm*AIH, violet); for clear comparison, orientation of at least one subunit from each superposition is the same as the orientation of *Mt*AIH. **(B)** Comparison of the secondary structure elements that form dimer interfaces of plant and bacterial AIHs (superposition of *Mt*AIH, blue, with *Hp*AIH, gray). Rectangles indicate zoomed regions; for clarity, part of the chains are semitransparent; the structure is rotated 150° with respect to the orientation in panel A.

In the PDB, there are several structures of bacterial representatives of AIHs that not necessarily form dimers like plant AIHs (Figure 6A). The bacterial AIHs form tetramers, like AIH from *Streptococcus mutans* (*Sm*AIH, PDB ID 2EWO) and *Enterococcus faecalis* (*Ef*AIH, PDB ID 2JER) (Llacer et al., 2007) or monomers like AIH from *Campylobacter jejuni* (*Cj*AIH, PDB ID 6B2W) (Shek et al., 2017). There are also dimeric bacterial AIHs like *Helicobacter pylori* (*Hp*AIH, PDB ID 3HVM) (Jones et al., 2010a) or *Porphyromonas gingivalis* (*Pg*AIH, PDB ID 1ZBR). *Hp*AIH and *Pg*AIH both present analogical dimer interface, however, it is different than the dimer interface of plant AIHs (Figure 6B). In these two bacterial dimeric AIHs, the interface residues are from repeats II and III. To be more precise, these residues correspond to the residues from β5, α4, α5, β6, and the loop between α7 and α8 of *Mt*AIH (Figure 4). This dimer interface of both bacterial AIHs is even smaller (~700 Å^2^, slightly above 5% of the monomer surface) than that of plant AIHs. A closer look at the superposition of *Mt*AIH with bacterial dimeric AIHs (Figure 6B, right panel) reveals the bacterial interface to be placed very close to the region of repeat II that in *Mt*AIH forms short helix α4. In plant AIHs, this region is important for the ligand binding and the conformation of α4 shows that it would create severe steric clashes with the dimer mate. These regions of bacterial dimeric AIHs and *Cj*AIH are five residues shorter. On the other hand, a closer look at the dimer interface of plant AIHs (Figure 6A, left panel) shows the different orientation of two helices in bacterial dimeric AIHs—η2/α2 and α5 with significantly different residues in these helices.

### Substrate Binding Mode of Plant AIHs

**Figure 7:**
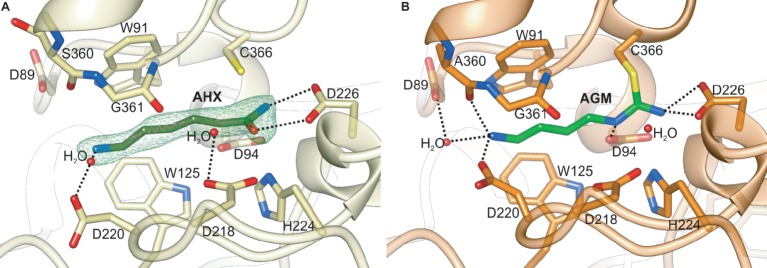
The catalytic site of plant AIHs. **(A)** Close-up view of the catalytic site of *Mt*AIH with bound AHX (dark green) in the chain C of the *Mt*AIH-AHX structure; green mesh represents Polder omit maps (contoured at 5σ) calculated in *Phenix* (Liebschner et al., 2017). **(B)** Imidine covalent intermediate of hydrolyzed AGM (lime green) captured in the crystal structure of *At*AIH (PDB ID 3H7H, unpublished structure). Dashed black lines indicate important hydrogen bonding interactions described in the main text.

The *Mt*AIH-AHX structure was obtained by cocrystallization and presents the bound AHX in three out of four protein chains that are present in the asymmetric unit. The ligand used for this study structurally differs from the physiological reaction product of *Mt*AIH, NCP, by the methylene which substitutes the secondary amine of NCP (adjacent to the carbamoyl moiety). Therefore, the conformation of the complex is similar to the conformation of the enzyme with the bound product after the reaction, representing a highly probable NCP binding mode (Figure 7A). It is worth to note that the *Mt*AIH-AHX structure shows a somewhat dynamic character where relative conformation of the ligand and surrounding residues (especially close to the terminal amine of AHX) is slightly different in each chain. Residues which are closer to the entrance of the active site have the B factor value significantly higher than the average B factor for the structure. The mean B factor value of the AHX (~35 Å^2^) is comparable to the structure average (~34 Å^2^), but it is still higher than the B factor of residues placed deep in the cavity. This can be explained by the nonphysiological character of AHX in comparison to AGM or even NCP; binding of the ligand was enabled due to its high concentration. The other available plant AIH structure which shows the details about the ligand binding mode is the structure of *At*AIH with the reaction intermediate (Figure 7B, PDB ID 3H7K, unpublished structure). Therefore, the substrate binding mode of plant AIHs can be described by the analogy of these two AIHs.

**Figure 8:**
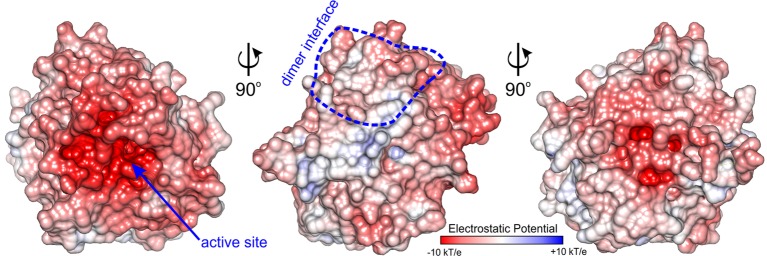
Charge distribution of *Mt*AIH. Surface electrostatic potential mapped on the surface of *Mt*AIH monomer calculated in *PDB2PQR* and *APBS* (Baker et al., 2001; Dolinsky et al., 2004); dimer interface is circled in blue.

The active site of *Mt*AIH is formed as a negatively charged channel (Figure 8) that is covered by coil region which links repeat IV and V (residues 291–314) which forms a kind of a lid over the catalytic site (Figure 2B). The active site itself is highly conserved among plant AIHs with the exception of Trp125 which in plant species can also be replaced by Tyr. Most likely, this does not drastically alter the shape and character of the channel. In the case of *Mt*AIH, the channel is formed by side chains of Trp91 and Trp125 where the planes of their indole rings are positioned almost perpendicularly to each other. The character of this region resembles the active site entrance of *Mt*CPA (Sekula et al., 2016), where also Trp residues shape the tunnel which guides to the catalytic Cys residue. In plant AIHs on the other side of the tunnel, there is Gly361 which due to the lack of side chain leaves the necessary void space for the ammonia and water molecules that are important for catalysis (see below for details).

Generally, GMEs bind their substrates with three different modes—1, 2A, 2B (Shirai et al., 2006). Of course, the bound substrates are structurally very diverse and also the orientation of the guanidine moiety placed in the vicinity of the catalytic triad is not always the same. In mode 1, substrates are bound in a way that their terminal parts (more distant from the catalytic triad) interact with residues from repeat IV and V. This promotes a completely different orientation of guanidine moiety at the bottom of the catalytic channel, which is rotated in comparison to the other two binding modes. The bound ligands in modes 2A and 2B interact with residues from repeat II and III. In the case of plant AIHs, the substrate binding mode corresponds to the mode 2 and is more similar to 2A, where the terminal amine group of AGM interacts with residues from repeat II. Therefore, the guanidine moiety of bound AGM reaches the active site bottom with a catalytic triad (in *Mt*AIH these are Cys366, Asp226, and His224) pointing toward Asp226. Polar residues that interact with the AGM guanidine moiety are Asn94, Asn226, and His224. They are responsible for the positioning of the plane of guanidine moiety very similar to the orientation of amide moiety of AHX (Figure 7A) so that it is susceptible to the nucleophilic attack from sulfur atom of Cys366 that is placed almost ideally on the line normal to the amide plane of AHX. The terminal amine of AGM placed by the entrance of the channel is stabilized by direct H-bonds with Asp220, Ala360, and a water-mediated H-bond with Asp89, analogous to the interactions observed in the *At*AIH structure (Figure 7B, PDB ID 3H7K, unpublished structure). Therefore, AGM that binds within the catalytic site of AIH is stabilized by a network of polar interactions involving every heteroatom in the substrate and by a series of hydrophobic interactions between its trimethylene moiety and the hydrophobic residues in the active site channel that connects the entrance with the catalytic site.

### Concerted Conformational Rearrangements Upon Ligand Binding

**Figure 9:**
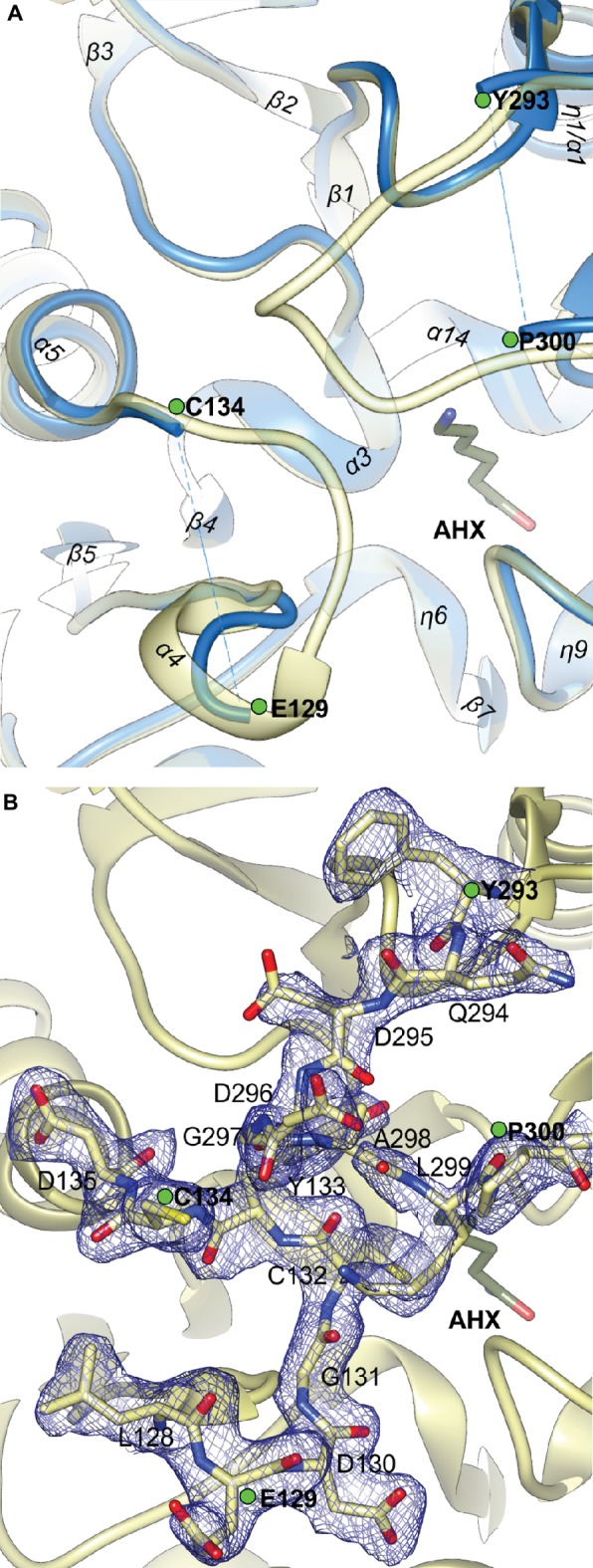
The gate-keeping loop of *Mt*AIH. **(A)** The superposition of non-liganded *Mt*AIH structure (blue ribbons) with the structure of *Mt*AIH-AHX complex (yellow semitransparent ribbons); green dots depict last visible residues from the loops 122–136 and 291–314 in the non-liganded *Mt*AIH structure. **(B)** Close-up view of the 2*F_o_–F_c_* electron density map contoured at 1σ (blue mesh) for the residues 128–135 and 294–300 in the chain C of *Mt*AIH-AHX structure.

The close vicinity of the *Mt*AIH active site is surrounded by two very flexible regions built mostly by long loops. One is the gate-keeping loop covering the active site (residues 291–314), which is a linker between repeat IV and V (Figure 2B). The other concerns the fragment 122–136 which belongs to the repeat II, where α4 is located. The gate-keeping loop is very variable in plant AIHs except for Arg301 and Arg306. Both regions are disordered in the non-liganded *Mt*AIH structure (Figure 9A), more precisely fragments between Glu129-Cys134 and Pro300-Tyr-293 were excluded from the structure due to the lack of electron density maps that would show their conformation. On the other hand, in the structure of *Mt*AIH-AHX complex, the electron density clearly shows their position (Figure 9B). This feature is also observed in *At*AIH (PDB ID 3H7K) with reaction intermediate, where the conformation of these coiled regions is fully modeled. Altogether, when the ligand is bound in the active site, these two regions come close together to form hydrogen bonds: between carbonyl oxygen of Cys132 and amide nitrogen of Lys299, and between backbone nitrogen of Cys134 and carbonyl oxygen of Gly297. Additionally, the guanidine group of Arg301 from the gate-keeping loop creates H-bonds with Asp220 and Asn35 in close vicinity of the AGM binding site. Therefore, this disorder-to-order transition secures the appropriate conformation of the bound substrate before reaction and the opening of the lid loop helps with product release after catalysis. The concerted disorder-to-order transition upon ligand binding was also observed in *Cj*AIH (Shek et al., 2017), however, it concerned different regions. More precisely, in *Cj*AIH, regions that showed conformational change upon substrate binding correspond to residues 122–136 and 212–224 of *Mt*AIH, therefore to the loops which are flanking α4 of repeat II and η9 which links repeats III and IV. The latter fragment in plant AIHs has a different sequence which, together with 18-residues shorter region of 278–314, results in the different recognition of the terminal amine of bound AGM in bacterial AIHs. Moreover, the sequence comparison of the plant AIHs suggests that the concerted conformational change of the gate-keeping coiled regions upon substrate binding can be characteristic for other plant AIHs as well. Likely, this feature can distinguish plant and bacterial orthologs, especially from those which present a shorter loop link between repeat IV and V.

### Cys366 Forms a Covalent Intermediate With AGM

The catalytic mechanism of guanidine-modifying enzymes is very similar to the cysteine proteases (Shirai et al., 2006). For AIHs it was structurally studied with bacterial orthologs (Llacer et al., 2007; Jones et al., 2010b) and involves the creation of a thioester covalent intermediate.

The reaction starts after binding of AGM when the gate-keeping loop is closed and the sulfur atom of Cys366 is ready to perform a nucleophilic attack on the central carbon of AGM guanidine moiety to form a tetrahedral covalent adduct. Then, His224 (positioned on the other side of the plane of amide moiety of AHX, Figure 7A) donates the proton to the closest amine of the intermediate, thus acting as a general acid for the reaction. This leads to the break of the adjacent bond and release of ammonia. Subsequently, ammonia is most likely H-bonded with the OD1 of Asp226 and it can be replaced by a water molecule (most likely the one, which is H-bonded with Asp218 in *Mt*AIH-AHX structure, Figure 9A) so the reaction can proceed. This water molecule is presumably moved deeper in the active site to be activated by transferring a proton to the His224/Glu226 charge relay network to form a hydroxide ion, so it can make a nucleophilic attack on the carbon of amidino intermediate to form another tetrahedral intermediate. The most probable position of the hydroxide ion which attacks the central carbon is represented by water in *At*AIH structure (Figure 7B). The intermediate collapses to form *N-*carbamoyl putrescine with a planar ureido carbon. The product of enzymatic action of AIH presents the conformation analogical to that of AHX (with an additional hydrogen bond with Asp94). Finally, the gate-keeping loops can be opened to release the product.

## Conclusions

The presented work described *Mt*AIH and compared its crystal structures with the other plant dimeric ortholog, AIH from *A. thaliana.* We have cross-validated our results with the reports on different plant AIHs highlighting residues that take part in the formation of AIH dimers in plants. These are residues from β2, β3, and η2/α2 from repeat I and α5 from repeat II. Plant AIHs are characterized by a different dimer interface to that observed in dimeric bacterial AIHs.

The crystallographic snapshots of *Mt*AIH together with deposited *At*AIH structures showed the detailed conformation of the coiled region that during the catalysis form a lid over the active site of plant AIHs. This loop is responsible for the recognition of the terminal amine of the bound AGM and provides necessary stabilization of the ligand in time of the catalytic event. Interestingly, the structural analysis of plant AIHs showed different disorder-to-order transition of the gate-keeping loops to that observed in bacterial orthologs, which shows that substrate recognition mechanism of plant AIHs differentiates them from bacterial AIH orthologs, especially those which present a shorter loop link between repeat IV and V.

## Data Availability

The datasets generated for this study can be found in Protein Data Bank, 6NIB, 6NIC.

## Author Contributions

BS planned and performed the experiments, analyzed the results, and wrote the manuscript. ZD analyzed the results and supervised the work.

### Conflict of Interest Statement

The authors declare that the research was conducted in the absence of any commercial or financial relationships that could be construed as a potential conflict of interest.

## Footnotes

^1^http://staraniso.globalphasing.org/cgi-bin/staraniso.cgi

## Abbreviations

ADC: Arginine decarboxylase
AHX: 6-aminohexanamide
AIH: Agmatine iminohydrolase
CPA: *N*-carbamoylputrescine amidohydrolase
GME: Guanidine-modifying enzyme
NCP: *N*-carbamoylputrescine
ODC: Ornithine decarboxylase
PA: Polyamine
PUT: Putrescine
SPD: Spermidine
SPM: Spermine.
